# AnimalAI: An Open-Source Web Platform for Automated Animal Activity Index Calculation Using Interactive Deep Learning Segmentation

**DOI:** 10.3390/ani15152269

**Published:** 2025-08-03

**Authors:** Mahtab Saeidifar, Guoming Li, Lakshmish Macheeri Ramaswamy, Chongxiao Chen, Ehsan Asali

**Affiliations:** 1 Institute for Artificial Intelligence, Franklin College of Arts and Sciences, University of Georgia, Athens, GA 30602, USA; mahtab.saeidifar@uga.edu; 2 Department of Poultry Science, College of Agricultural and Environmental Sciences, University of Georgia, Athens, GA 30602, USA; sean.chen@uga.edu (C.C.); ehsanasali@uga.edu (E.A.); 3 School of Computing, Franklin College of Arts and Sciences, University of Georgia, Athens, GA 30602, USA; laksmr@uga.edu

**Keywords:** animal behavior, deep learning, software, activity index

## Abstract

Monitoring the activity index of animals is crucial for assessing their welfare and behavior patterns. However, traditional methods for calculating the activity index, such as pixel intensity differencing of entire frames, are found to suffer from significant interference and noise, leading to inaccurate results. These classical approaches also do not support group or individual tracking in a user-friendly way, and no open-access platform exists for non-technical researchers. This study introduces an open-source web-based platform that allows researchers to calculate the activity index from top-view videos by selecting individual or group animals. It integrates Segment Anything Model2 (SAM2), a promptable deep learning segmentation model, to track animals without additional training or annotation. The platform accurately tracked Cobb 500 male broilers from weeks 1 to 7 with a 100% success rate, IoU of 92.21% ± 0.012, precision of 93.87% ± 0.019, recall of 98.15% ± 0.011, and F1 score of 95.94% ± 0.006, based on 1157 chickens. Statistical analysis showed that tracking 80% of birds in week 1, 60% in week 4, and 40% in week 7 was sufficient (r ≥ 0.90; *p* ≤ 0.048) to represent the group activity in respective ages. This platform offers a practical, accessible solution for activity tracking, supporting animal behavior analytics with minimal effort.

## Simple Summary

Understanding how animals move is important for ensuring their health and well-being. However, traditional methods used to measure animal activity are often inaccurate, difficult to use, and not accessible to those without technical skills. In this study, we developed a free, easy-to-use online tool that allows researchers to measure animal activity using video footage. Users can simply upload a video and click on the animals they want to track. Then the system automatically follows the animals and calculates how active they are. This tool was tested on broiler chickens and showed very accurate results, even when the animals were at different ages or in varied lighting conditions. Unlike older methods, this tool focuses only on the animals and ignores interference in the background, such as moving people or equipment, which improves accuracy. It also works without requiring any programming knowledge or complicated setup. By making movement tracking more accurate and accessible, this tool can help researchers, farmers, and animal care professionals monitor animals more effectively and make better decisions to support automatic animal behavior analytics.

## 1. Introduction

Animal activity plays a pivotal role in understanding welfare, health, and behavior patterns across various livestock species [1,2,3]. In modern animal production systems, continuous observation and prompt detection of abnormal behaviors are paramount for maintaining high standards of welfare and maximizing productivity [4]. Capturing animal activity, broadly defined as the frequency or extent of movement over time, can offer valuable insights for both researchers and producers to make evidence-based decisions. For example, in poultry, sudden changes in flock movement may indicate issues like heat stress or disease outbreaks [2]. In cattle and pigs, activity patterns can help detect lameness or identify periods of increased stress [5,6,7]. As such, techniques that enable robust and efficient estimation of the activity are indispensable.

In order to quantify animal activity, the activity index, a measure of movement intensity through image processing, was proposed by [8]. The activity index was defined as the percentage of pixels of moving objects to the total number of pixels within the image (including animals and background). In more recent research, the total number of pixels was replaced with total bird-representative pixels to compensate for variations in animal size at different ages [9,10,11]. Since the concept was coined, the activity index has been widely used to quantify the activities of broilers [12,13]. The concept has been applied to develop a commercial computer vision system, named eYeNamic, and the vision system has been applied in several European studies [11,14].

While the classical activity index calculation method can be quick to implement and computationally straightforward, it tends to be highly sensitive to noise and environmental factors such as lighting fluctuations, camera vibrations, or background movements such as human interference [15]. Moreover, applying pixel intensity differencing to an entire scene restricts researchers to group-level activity assessments. In many practical scenarios, especially those involving large populations of animals housed together, the interest lies in pinpointing the movements of specific individuals. Without the ability to segment and track individual animals, vital data, such as determining which animals are underactive or hyperactive, remains inaccessible.

Deep learning-based methods have substantially advanced object detection and segmentation in recent years [16,17,18,19,20]. A recent review concludes that the YOLO family has become a technological cornerstone for intelligent animal phenotyping, with demonstrated applications spanning body-size estimation, individual identification, behavior monitoring, and biomass estimation [21]. In another study, a camera-based system using deep learning detection (0.98 precision and 0.90 recall) and tracking (0.75 accuracy) clustered broiler movements into ‘least active,’ ‘active,’ and ‘highly active,’ showing strong potential for automated welfare monitoring in commercial farms [22]. However, developing a specialized segmentation model for each livestock species or experimental setup can be prohibitively time-consuming and expensive. Researchers would need to curate and annotate large image datasets, train custom deep learning models, and then continuously update these models as lighting conditions, camera angles, or animal growth stages change. This complexity has motivated the rise in more generalized segmentation models that are pre-trained on vast and diverse image corpora, allowing them to perform “zero-shot” or “few-shot” segmentation on new types of objects [23]. One such model is Segment Anything Model 2 (SAM2), a powerful variant of the foundational SAM [23]. SAM2 has been lauded for its ability to quickly and accurately identify objects of interest with minimal prompting, effectively reducing the need for large-scale annotation [23]. Unlike traditional models that often fail when confronted with new species or environments, SAM2 has been broadly trained with billions of image masks, enabling it to handle a wide range of scenes and animal morphologies [17,24,25,26]. Alongside these advances in segmentation, there has also been a growing need for accessible, user-friendly platforms that can seamlessly integrate deep learning into everyday research workflows. The user-friendly platforms are especially important for scholars who do not have sufficient computing backgrounds for coding but would love to use the automatic tools to support animal research for advancing animal products.

Several user-friendly platforms were developed in the animal behavior domain to assist researchers in tracking and analyzing animal movements. For instance, the AnimalAccML integrated multiple machine learning models and feature engineering techniques and enabled users to automatically analyze behaviors with several mouse clicks based on triaxial accelerometer data, which is not suitable for computer vision-based metric analytics [16]. DeepLabCut was a widely adopted open-source tool that leverages deep learning for markerless pose estimation in images/videos [27]. Its user-friendly interface made it popular among researchers; however, it generally required extensive manual annotation and a considerable amount of training data to adapt to different species or experimental conditions. This reliance on manual setup hinders rapid deployment in novel environments and limits its utility for studies that require efficient analysis. Another notable example is idtracker.ai, which offered automated tracking of individual animals within groups [28]. While it simplified the tracking process and is relatively intuitive, idtracker.ai tended to be computationally intensive, especially when dealing with large groups or high-resolution video footage. Moreover, its performance degraded in scenarios with significant noise, variable lighting, or complex backgrounds, thereby reducing its reliability in accurately capturing animal movement dynamics [29].

Despite the advancements these platforms represent, they were not designed to compute the animal activity index automatically. Their primary focus lies in detailed tracking and pose estimation rather than providing a comprehensive, user-friendly solution for calculating movement-based metrics such as the activity index at either the individual or group level. In conclusion, while current tools offer valuable functionalities in animal tracking and behavior analysis, there remains a notable gap in a user-friendly platform that automatically calculates the animal activity index, highlighting an unmet need in animal welfare research and monitoring.

Several studies in the field of collective animal behavior have demonstrated that monitoring a representative subset of individuals can effectively capture the overall dynamics of a group, aiming to improve computational efficiencies. For example, in a study investigating the spatial organization and interaction rules within starling flocks, researchers found that each bird interacted with a fixed number of neighbors (six to seven) rather than all nearby individuals. This topological interaction enabled flocks to maintain cohesion and coordinated movement, even under changing densities and external perturbations. Although the study did not directly address representative sampling, the idea that a limited number of local interactions govern the behavior of the entire group implies that monitoring a subset of individuals could reveal key aspects of collective dynamics [30]. Similarly, in another study on the collective behavior of midge swarms, researchers found that individual midges were strongly connected, even beyond their nearest neighbors. Even in the absence of global order, midges exhibited coherent movement patterns that could be explained by localized interactions. Their study demonstrated that these correlations reflect emergent group-level behavior, suggesting that sampling a fraction of individuals can provide reliable insights into the overall dynamics of the swarm. By employing simulations of interacting particles, they further showed that local measurements could scale up to describe the collective response of the entire group [31].

Our exploration of different sampling ratios (20%, 40%, 60%, and 80%) across key growth stages in broilers addressed this gap. By systematically determining the optimal proportion of birds needed to accurately represent the entire flock’s activity, our study provides a practical framework that reduces computational demands without compromising the reliability of activity index measurements. This tailored approach is particularly relevant for commercial applications, where rapid and resource-efficient monitoring is essential for effective animal behavior analytics. The objectives of this research were to (1) develop a user-friendly, open-source platform to enable researchers to calculate the activity index of animals, either individually or in groups, from video footage; and (2) explore the representative proportion of animals to depict the whole group activity index, to save computing time and resources.

## 2. Materials and Methods

### 2.1. Animal Housing and Video Data Collection

For validating the segmentation model, a subset of a larger video dataset was used. This dataset was collected at the University of Georgia’s Poultry Research Center during May–June 2024. A total of 1776 day-old Cobb 500 male broiler chickens were randomly assigned to 48 pens, with 37 birds being allocated per pen, within two environmentally controlled rooms. The rooms were measured to be approximately 17.2 m in length by 11.4 m in width and were subdivided into two rows of 12 identical pens, each of which measured 1.2 m by 3.0 m. Two feeders were provided at opposite ends of every pen, and two centrally located drinking lines were installed. Standard environmental conditions were maintained in accordance with the Cobb management guidelines (Cobb, 2022 [32]), with feed and water provided ad libitum. Lighting and temperature adjustments were made according to age-specific protocols throughout the rearing period. Video recordings were acquired using overhead security cameras (NHD-887MSB, Swann Security, Santa Fe Springs, CA, USA) that were mounted on the ceiling at approximately 3.05 m above each pen. Continuous recordings were managed by 16-channel video recorders (SRDVR-85680H-US, Swann Security, Santa Fe Springs, CA, USA). The recordings were set at a resolution of 1024 × 768 pixels and at 15 frames per second (fps), and the video data were stored as MP4 files on a 20-terabyte external hard disk. A total of 34 videos from week 1 through week 7 were selected, and 1157 individuals were used for evaluation. Although all birds from the large study were included in the complete dataset, the subset for evaluation was chosen to ensure a representative distribution across developmental stages from week 1 (early phase), week 4 (medium phase), and week 7 (late phase). All experimental procedures, including the video recordings, were performed in compliance with protocols approved by the Institutional Animal Care and Use Committee (IACUC) at the University of Georgia (protocol number: A2023 07-016-Y1-A0).

### 2.2. Overall Workflow

**Figure 1:**
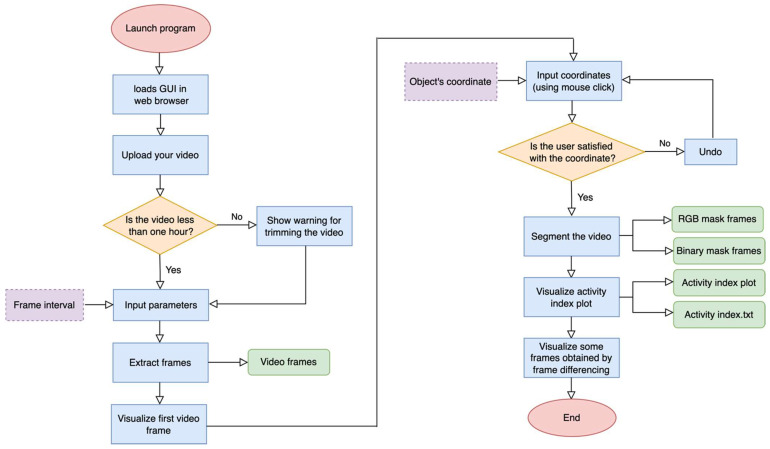
Workflow diagram of the platform. This figure presents a schematic of the different steps analytical process employed in the platform. Red color indicates start and end points of the process; blue color indicates main processing steps; orange color indicates decision points; purple color indicates user input parameters; and green color indicates files saved in the main directory.

Figure 1 illustrates the Streamlit-based interface workflow for calculating the animals’ activity index. Once the application was launched, a user-friendly graphical interface was loaded in the default web browser. The user could upload a video of up to one hour in length for the convenience of data visualization. If a video exceeded this duration, the interface issued a warning and recommended trimming. Subsequently, key parameters, such as frame interval, can be specified by the user. The system extracted frames from the uploaded video and displayed the first frame so the user can pinpoint, via mouse click, the location of the animal or region of interest. If the user was dissatisfied with the selected coordinate, an ‘undo’ option reverted the choice until the coordinate was precisely defined. After confirming the chosen coordinates, the interface proceeded to segment the video, generating both an RGB mask frame and a binary mask frame. This segmentation underpinned the computation of an activity index, which was then plotted and viewable within the interface. Additionally, the activity index plot, as well as the normalized activity index for each consecutive frame, were saved as a PNG and a TXT file, respectively. In addition, users can inspect frames derived from frame differencing for a more detailed overview of movement and check whether the segmentation was successful or not. Although Figure 1 shows a typical workflow, users may adjust certain steps (e.g., re-uploading trimmed clips or revisiting parameter settings) according to their experimental needs. Detailed descriptions of each phase and the options offered by the Streamlit interface are provided in the following sections. The interface was published on GitHub (https://github.com/MahtabSaeidifar/AnimalAI, version 1.0, accessed on 1 June 2025) for open access.

In this study, the entire platform was developed solely using Python 3.10, which enabled all components to be consolidated into a single consistent computing environment to enhance code readability and maintainability. The most important packages used in our platform were torch (v2.4.1) for deep learning, Streamlit (v1.19.0) for developing interactive web applications, numpy (v1.26.4) for numerical computations, pandas (v1.4.2) for data manipulation, matplotlib (v3.9.2) for data visualization, and jupyterlab (v4.2.4) for providing an interactive development environment. In addition, the SAM2 package was installed directly from its GitHub repository (https://github.com/facebookresearch/sam2, accessed on 1 June 2025) to facilitate segmentation tasks. The computer used for platform development and evaluation was a Dell Precision workstation (Dell Inc., Round Rock, Texas, USA) equipped with a 13th Gen Intel^®^ Core™ i7-13700 processor, featuring 24 logical CPUs with clock speeds ranging from 0.8 GHz to 5.2 GHz, 62 GiB of installed RAM, and a 64-bit operating system.

### 2.3. Video Uploading

Once the application was launched, a user-friendly graphical interface was displayed in the web browser, allowing for an intuitive interaction. The interface prompted the user to upload a video, accepting various formats (e.g., MP4, MOV, AVI, and MPEG4). The recommended maximum duration for the video was one hour; if the uploaded file exceeded this length, the system automatically issues a warning and advises trimming the video to under one hour. This recommendation helps ensure faster processing times and reduces computational overhead during subsequent steps. The system does not transmit, store, or share uploaded videos to any external database or third-party server. This ensures that all data, including the uploaded videos, remains fully private and under the user’s control.

### 2.4. Video Frame Extraction

Once the user has uploaded a video, the application automatically evaluates the duration of the file and generates a range of recommended frame intervals. These recommendations aimed to strike a balance between capturing sufficient details and minimizing both storage requirements and computational resources. While users are free to override the recommended settings and specify a custom interval, adhering to the suggested range is generally preferred for optimal efficiency and data manageability.

**Figure 2:**
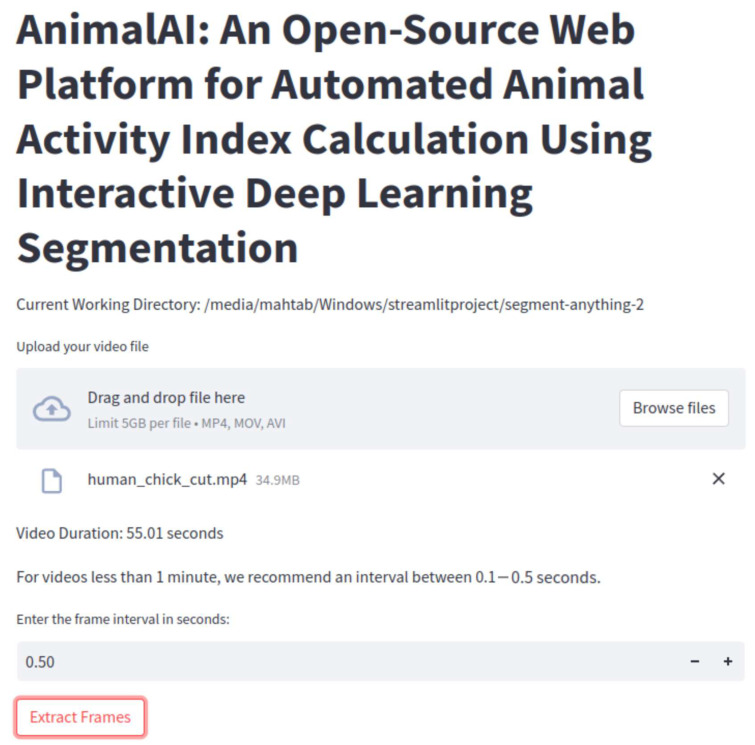
The graphical user interface of the application displays the frame extraction process. Users can select a frame interval after uploading a video, adjust settings based on recommendations, and trigger the extraction process using the ‘Extract Frames’ button.

By selecting an interval, the user essentially controlled the frequency of frames to be extracted: smaller intervals yield more frames (allowing for finer-grained analysis) but require greater storage and computational power, whereas larger intervals reduce the number of frames extracted and offer lowered storage demands at the potential cost of missing some subtle movements. After choosing a frame interval, users can click the ‘Extract Frames’ button to trigger the extraction process, as shown in Figure 2. The resulting frames were automatically stored in a designated directory, ensuring that they can be readily accessed in subsequent stages of the workflow (e.g., segmentation, activity index calculation, or further analysis).

### 2.5. Interactive Animal Selection

Following frame extraction, the interface automatically displayed the first frame from the video so that the user could identify the animal(s) to be segmented from the background. Using a mouse click, users can select one or multiple animals (e.g., one, two, three, or potentially all visible objects) within the frame. Each click isolated the chosen subject by registering its coordinates, which guide subsequent segmentation tasks. If the user is dissatisfied with any selections, an ‘undo’ button enables a quick reversion, allowing for precise, iterative refinement of the selected coordinates.

This interactive step was crucial for achieving reliable isolation of the target animals from extraneous background elements. By removing other moving objects and environmental noises, the application is better able to deliver an accurate analysis of movement or behavior in subsequent phases. Moreover, the flexibility to select multiple animals within a single frame offers a comprehensive approach for studies involving group dynamics or interactions.

### 2.6. Segmentation Using Segment Anything Model 2

**Figure 3:**
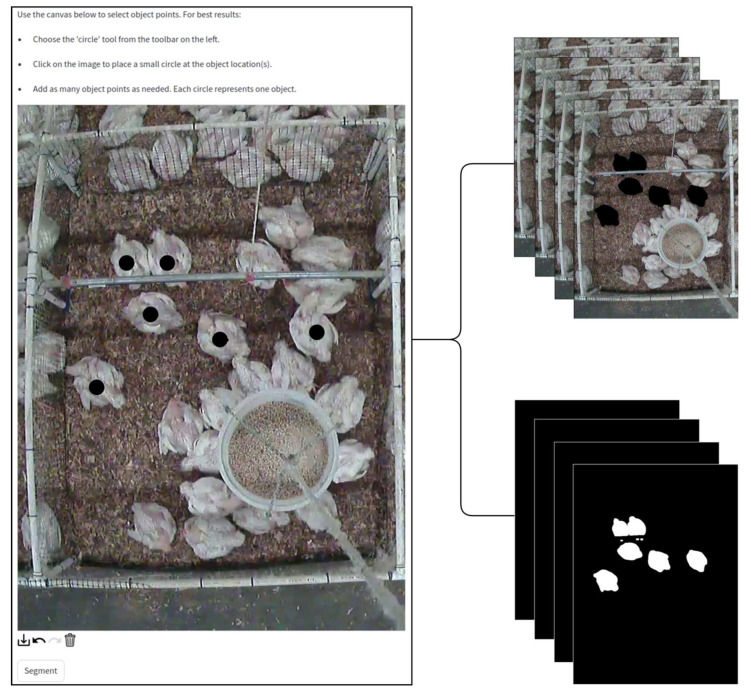
Examples of segmentation outputs generated by the application using the Segment Anything Model 2. The RGB mask frames (**top-right**) highlight the selected animals in distinct colors, while the binary mask frames (**bottom-right**) isolate the targeted animals from the background.

Once the targeted animals were selected, the segmentation process was initiated by clicking on the ‘Segment’ button (Figure 3). The foundation model known as SAM2 was employed to handle promptable visual segmentation in both images and videos. In SAM2, a data engine was built and refined through user interactions, culminating in the creation of the largest video segmentation datasets to date. A simple transformer architecture with streaming memory was adopted to enable real-time video processing [23].

By leveraging its extensive pretraining on a large and diverse dataset, SAM2 demonstrated strong performance across a wide range of segmentation tasks in both videos and images. In the context of video segmentation, higher accuracy has been observed with only one-third the user interactions required by previous approaches, and image segmentation ran 6× faster and more accurately compared to the original SAM. Notably, no additional training was required for specific tasks; instead, the user-selected coordinates served as prompts for guiding the segmentation, which was then automatically propagated to subsequent frames.

After the segmentation process was completed, two directories were created to store the results. One directory housed the RGB mask frames, in which the selected animals were distinctly highlighted, while the other stored the binary mask frames, where only the targeted animals were shown in isolation. Figure 3 illustrates examples of both the RGB mask frames and the corresponding binary mask frames.

### 2.7. Frame Differencing for Calculating Activity Index

**Figure 4:**
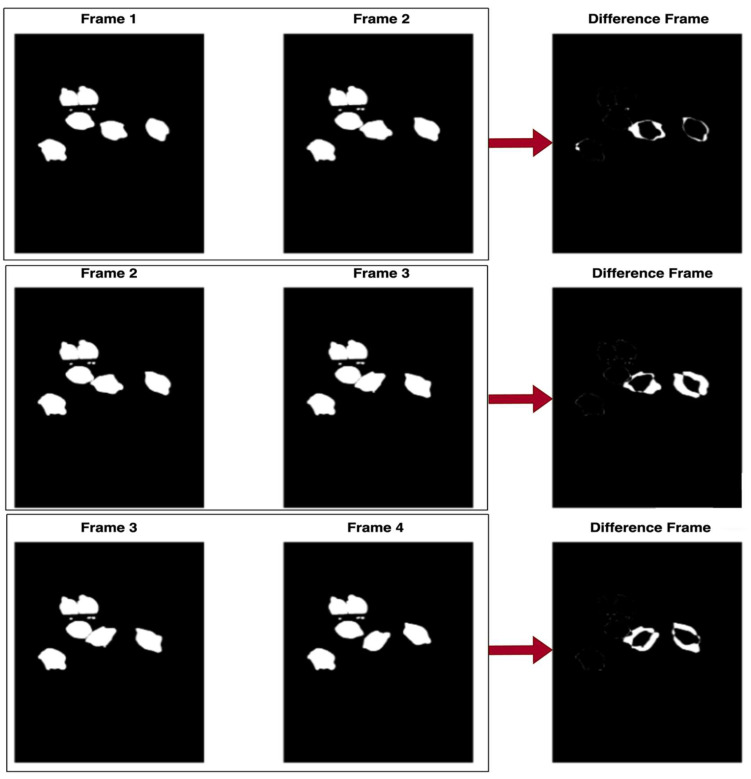
A series of differenced frames illustrating pixel-level changes between consecutive segmented frames (binary mask frames) for calculating the animal activity index.

Activity within a video sequence was assessed by measuring the extent of pixel-level changes between consecutive segmented frames (i.e., the binary mask frames). To achieve this, the difference between the current binary frame and the preceding frame was computed using an absolute difference operation. The resulting differenced frame highlights any pixels that have changed, indicating movement or behavioral changes. Figure 4 illustrates a series of these differenced frames.

To obtain an overall measure of activity, the number of changed pixels in each differenced frame was normalized by the combined pixel count of the current and previous frames. Formally, the activity index for the frame i is calculated in Equation (1).

(1) $${Activity\;index}_{i}=\frac{{Diff\text{\_}pixel\text{\_}count}_{i}}{Pixel\text{\_}count\text{\_}current+Pixel\text{\_}count\text{\_}previous}$$

where the term ${Diff\text{\_}pixel\text{\_}count}_{i}$ is the total number of nonzero pixels in the “difference frame,” which is obtained by subtracting the pixel values of frame i+1 from frame i. Thus, these nonzero pixels highlight the regions that have changed between the two consecutive frames. Meanwhile, Pixel_count_current and Pixel_count_previous each represent the total number of nonzero pixels in frames i+1 and i, respectively. The two frames were used to generate the difference frame. This ratio ensured that the activity index remained bounded between 0 and 1. A higher value indicated greater movement, while a lower value suggested minimal changes.

The rationale for normalizing by the sum of the pixel counts from two consecutive frames is to ensure that the calculated activity index remains bounded between 0 and 1, even in cases of extreme movement. For example, consider two consecutive frames where the animal’s location is completely different with no overlap—such as when the animal makes a sudden jump or moves at very high speed. In the difference frame, both positions of the animal (from frame 1 and frame 2) appear as masks. By dividing by the sum of the pixel counts of both frames, the activity index becomes 1 (the maximum), correctly representing this extreme movement. If we were to normalize using only one frame’s pixel count, the calculated activity index could exceed 1 in such scenarios, which would be mathematically incorrect and misleading. This normalization approach also reduces the impact of variations in the visible area of the animals caused by posture changes, partial occlusion.

Additionally, the normalized activity index for each consecutive frame was saved in a TXT file in the main directory. This is useful for users to further analyze the results on their own, enabling deeper insights into movement patterns and behavioral trends from an animal scientist’s perspective.

### 2.8. Visualizing the Activity Index

**Figure 5:**
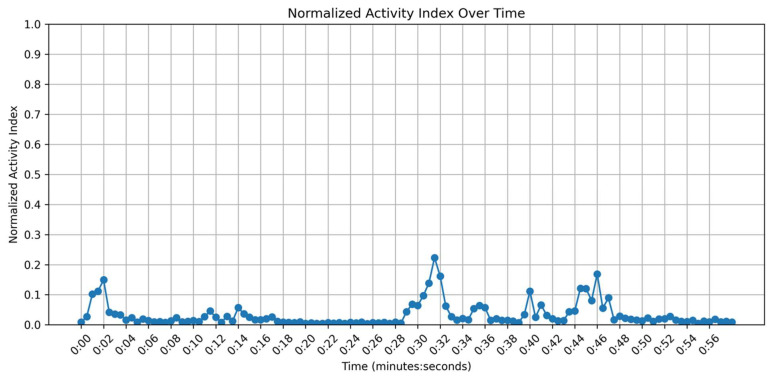
Activity index plot illustrating the level of movement for the selected animals throughout the video. The *x*-axis denotes the video duration in minutes and seconds, while the *y*-axis ranges from 0 (minimal activity) to 1 (maximum activity).

Once the frame differencing procedure was completed, an activity index plot was automatically generated to illustrate the level of movement for the selected animals throughout the video. As shown in Figure 5, the *x*-axis represents the video time in minutes and seconds, while the *y*-axis ranges from 0 (indicating no movement) to 1 (reflecting the highest activity index). For each timestamp, a corresponding activity index value was displayed, enabling researchers to identify periods of heightened activity or relative inactivity. This visualization was invaluable for understanding the dynamics of animal behavior, as it condensed movement data into a single, intuitive plot for efficient analysis. Additionally, the generated activity index plot was saved in the main directory, allowing users to access and utilize it for further examination or reporting.

### 2.9. Evaluation Metrics Calculation

This study employed a robust suite of evaluation metrics to independently gauge the performance of tracking and segmentation. The SAM2 evaluation leverages a dataset consisting of 1157 individual chickens from 82 different video frames. The annotation of these images was carried out by a well-trained technician using Roboflow, which ensured the provision of high-precision masks that delineated the most complete depiction of each chicken in each frame. Subsequently, another well-trained technician conducted a double verification to guarantee the accuracy and quality of the labeling. This rigorous ground truth formed the benchmark for assessing the segmentation models’ accuracy.

The SAM2 segmentation performance was evaluated with *precision*, *recall*, *F1 score*, and *Intersection over Union* (*IoU*) as described in Equations (2)–(5). The *precision* measures the accuracy of the segmentation model in identifying only relevant pixels as part of the segmentation. It is the ratio of correctly predicted positive observations to the total predicted positive observations. *Recall*, also known as sensitivity, measures the model’s ability to correctly identify all relevant pixels. It is the ratio of correctly predicted positive observations to all observations that should have been labeled as positive. The *F1 score* is the harmonic mean of *precision* and *recall* and a measure of the model’s accuracy. An *F1 score* reaches its best value at 1 (perfect *precision* and *recall*) and worst at 0. *IoU* is a measure used to quantify the percent overlap between the target mask and the model’s prediction output. It is calculated by dividing the area of overlap between the predicted segmentation and the ground truth by the area of union.

(2) $$Precision=\frac{True\;positive}{True\;positive+False\;positive}$$

(3) $$Recall=\frac{True\;positive}{True\;positive+False\;negative}$$

(4) $$F1\;score=2\times \frac{Precision\times Recall}{Precision+Recall}$$

(5) $$IoU=\frac{True\;positive}{True\;positive+False\;positive+False\;negative}$$

where True positive refers to pixels that are correctly identified as part of birds; False positive are the pixels that the segmentation model incorrectly identifies as part of birds, but they actually belong to the background; False negative is used for pixels that are part of birds in the ground truth but are missed by the segmentation model.

A successful segmentation is one where the *IoU* is 50% or greater, which aligns with standard thresholds used in prominent publications [33,34,35]. The *success rate* thus reflects the percentage of images in which the models successfully tracked and segmented the chicken areas shown in Equation (6).

(6) $$Success\;rate=\frac{Number\;of\;successfully\;tracked\;\&\;segmented\;images\;(IoU>0.5)}{Total\;number\;of\;images}$$

### 2.10. Evaluating the Impact of Segmentation on Activity Index Accuracy

To evaluate whether segmentation improved the accuracy of the activity index, 480 video frames were selected from week 4 recordings. These frames contained human interference and other unnecessary object movements (e.g., feeders and fans), providing a challenging scenario for activity-index calculation. Two methods were applied: First, the conventional “no-segmentation” approach involved subtracting consecutive frames to generate a difference frame, followed by applying a threshold value of 50 to binarize the result. White pixels in the binary image indicated movement, and black pixels indicated no movement. The activity index for each frame was then calculated based on the count of white pixels in that frame.

Second, in the segmentation-based method, all chickens in each frame were isolated using SAM2 before frame differencing. This removed non-essential background elements, including any human interference. The white-pixel counts were again used to compute the activity index. To determine whether these two approaches (with and without segmentation) produced significantly different mean activity levels, a paired *t*-test was conducted, with statistical significance set at *p* < 0.05. This comparison enabled a clearer assessment of how removing background motion influences the reliability of activity-index measurements.

### 2.11. Statistical Analysis of Different Ratios of Birds to Represent the Entire Group’s Activity

Tracking every individual bird can be time-consuming and computationally expensive. Consequently, this study tested whether sampling a subset of birds could reliably represent the entire flock’s movement patterns at different growth stages. Four different sampling ratios of 20%, 40%, 60%, and 80% of the flock were compared to the 100% baseline at three ages (weeks 1, 4, and 7). The number of birds selected from a pen was 7 for 20%, 15 for 40%, 22 for 60%, 30 for 80%, and 37 for 100%. Six distinct initializations (i.e., sets of randomly selected birds in feeder, drinker, corner, and open regions of the pen) were used per ratio to reduce spatial bias. All video data for this analysis were obtained, as described in Section 2.1. Briefly, from each selected video clip, 480 consecutive frames (15 frames per second over ~32 s) were extracted. Within these frames, the developed platform isolated only the chosen subset of birds for each ratio, and an activity index was calculated by comparing pixel-wise differences between consecutive segmented frames. Parallel calculations were made for the 100% baseline (i.e., the entire flock).

The Pearson correlation coefficient (*r* value), as shown in Equation (7), was computed between each subset’s activity index (at varying sampling percentages) and the full flock’s index across six different initializations. The analyses were performed in Python (v3.9) using the pandas, numpy, and statsmodels libraries. This approach enabled a straightforward evaluation of whether a reduced sampling ratio could reliably represent overall flock activity while minimizing computational overhead.

(7) $$r=\frac{\sum \left(x_{i}-\bar{x}\right)\left(y_{i}-\bar{y}\right)}{\sqrt{\sum {\left(x_{i}-\bar{x}\right)}^{2}{\sum \left(y_{i}-\bar{y}\right)}^{2}}}$$

where $x_{i}$ represents the $i^{th}$ observation for variable X, $y_{i}$ represents the $i^{th}$ observation for variable Y, $\bar{x}$ is the mean of all X values, and $\bar{y}$ is the mean of all Y values. The numerator captures how X and Y co-vary (or change together), while the denominator normalizes these deviations, keeping r dimensionless and ranging from −1 to +1.

According to a study, the correlation was negligible with r being 0.00 to 0.30 or 0.00 to −0.30, low with r being 0.31 to 0.50 or −0.31 to −0.50, moderate with r being 0.51 to 0.70 or −0.51 to −0.70, high with r being 0.71 to 0.90 or −0.71 to −0.90, and very high with r being 0.91 to 1.00 or −0.91 to −1.00 [36]. Additionally, following the computation of the r value between each representation and the entire flock, a statistical comparison of the activity indices was carried out across different pairs of representations to determine whether they differ significantly. A significance level of *p* < 0.05 was applied, meaning that any *p*-value below 0.05 indicates a significant difference, while values above this threshold suggest no meaningful difference. If no statistically significant differences were observed, a smaller sampling ratio may be selected without sacrificing accuracy, thus reducing both computational load and resource requirements.

## 3. Results and Discussion

### 3.1. Example Procedure of Interface Operations

Below is a general procedure for video-based activity index calculation using the developed web-based platform. The platform guided users step-by-step through video segmentation and activity index generation. Some of the computational user interfaces are presented in Figure 2, Figure 3, Figure 4 and Figure 5.

Step 1: Run the platform and launch the interface using the command, which loads the platform in a web browser. The main interface page then appears one a default web browser.

Step 2: Click the ‘Browse files’ button to upload a video file.

Step 3: The platform automatically checks whether the uploaded video is less than one hour in duration. If it is not, a warning message will be displayed, prompting the user to trim the video before proceeding.

Step 4: Input the frame interval for frame extraction based on either the recommended frame interval or the user’s choice.

Step 5: Once the frame interval is set, the platform will extract individual frames from the video. The first frame will be displayed for visualization and selection of the animals to be tracked.

Step 6: Using the mouse, the user can click on the animal’s location within the first frame to input its coordinates. This step initializes the segmentation process by identifying the region of interest.

Step 7: Confirm whether the selected coordinates are correct. If not, click the ‘undo’ button to adjust the coordinates and select a new region.

Step 8: When satisfied with the input, click the ‘Segment’ button. The platform will begin segmenting the video, generating both RGB mask frames and binary mask frames that highlight the animals of interest.

Step 9: The platform displays the activity index plot for the targeted, segmented animal across the video.

Step 10: Finally, the platform displays frames obtained through frame differencing, providing a dynamic view of motion changes throughout the video.

### 3.2. Segmentation Performance on a Chicken Dataset

**Figure 6:**
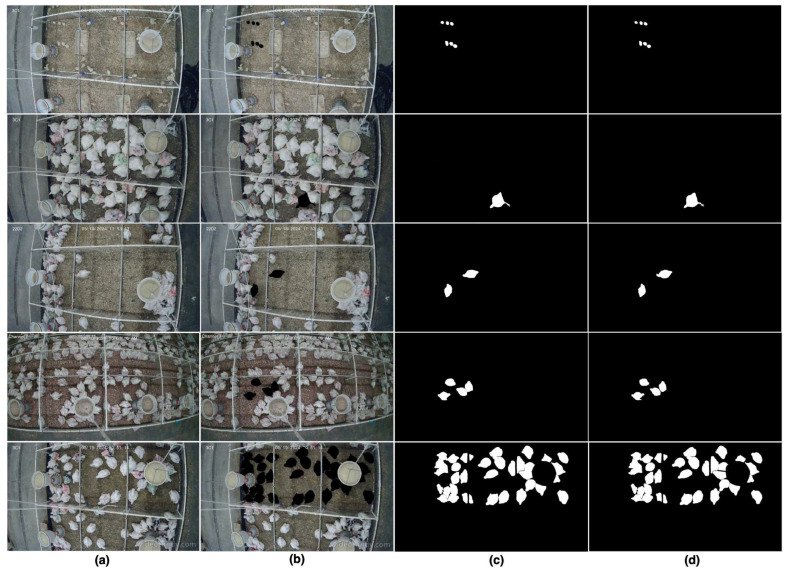
SAM2 segmentation results: (**a**) the original frame, (**b**) the corresponding RGB mask output, (**c**) the binary mask output, and (**d**) the ground truth segmentation.

In this study, the effectiveness of SAM2 was evaluated within a web-based pipeline using a dedicated chicken dataset as mentioned in Section 2.1. The dataset comprised multiple video clips captured under diverse lighting conditions (5–10 lux), varying stocking densities (30–37 birds in a 1.2 m wide × 3.0 m long pen), and different chicken ages (weeks 1 to 7). This enabled us to challenge the model’s robustness under realistic, real-world scenarios. As shown in Figure 6, SAM2 produces high-quality segmentation results across chickens of different ages, demonstrating its adaptability to variations commonly encountered in poultry management settings.

SAM2 was selected for this project because it is specifically designed for interactive, prompt-based segmentation. In practice, a user can indicate the animal of interest by simply clicking or drawing a bounding box, after which SAM2 automatically tracks and segments that animal throughout the video. This user-driven workflow was ideally suited for a web-based application where videos were uploaded, the target object(s) were selected, and precise mask outputs were generated without reliance on a fixed set of predefined object classes. By using prompts, the model effectively mitigated challenges posed by occlusions and cluttered backgrounds, which are the issues frequently encountered in livestock environments. This approach was consistent with earlier studies that have shown minimal, yet precise user input, which can substantially improve segmentation accuracy [37,38].

To quantify the segmentation accuracy of SAM2 on chickens at various ages, several established performance metrics, including *precision*, *recall*, *F1 score*, *IoU*, and *success rate*, were employed. Table 1 summarizes these quantitative results for segmenting broiler chickens in weeks 1, 4, and 7. The consistently high scores (100% *success rate*, over 92% *precision*, over 97% *recall*, over 92%, and over 90% *IoU*) across different conditions indicate that SAM2 can generalize well, even when the visual appearance of the subjects changes due to factors such as age or lighting. It is worth noting that chickens present a unique segmentation challenge since they are uniformly white and look very similar to each other, with less defined boundaries compared to other species, such as pigs or cows, that have distinct markings and clearer shapes. Despite these challenges, the model maintained high performance throughout. However, it is important to clarify that the platform is designed for environments where animals are kept inside a pen or a bounded area that is fully captured by the camera frames, as was the case in our study with chickens in a pen, to ensure that no animals leave the frame during tracking. Earlier segmentation methods relied on user-drawn bounding boxes or scribbles and can struggle with background clutter and occlusion [39]. Unlike the earlier methods, the current method leverages prompt-based guidance to focus precisely on regions of interest with the robust model architecture. Furthermore, SAM demonstrated high efficiency in practical deployment, requiring minimal user interaction while achieving accurate segmentation. Its refined prompt-based strategy effectively directed the model’s attention to relevant regions, enabling precise segmentation without extensive manual annotation.

The demonstrated performance has clear implications for real-world applications in precision poultry monitoring. For instance, integrating SAM2 into a web-based system would allow research scholars, regardless of coding or computing expertise, to upload videos, use simple prompts to segment individual chickens, and receive accurate segmentation masks in real time. Such a system would not only facilitate automated flock monitoring and behavioral analysis but could also be extended to support tasks such as weight prediction or movement tracking. Recent advancements in poultry monitoring have further illustrated how segmentation outputs can be utilized as critical inputs for data-driven livestock management. For instance, SAM-segmented results were combined with thermal images to extract various statistics of chickens’ body temperature, facilitating more accurate assessments of their thermal conditions [17].

Overall, the robust performance of SAM2 across diverse environmental and biological conditions, combined with its interactive and user-friendly design, confirmed its suitability for applications that require high-quality segmentation with minimal manual input. These results validate the technical capabilities of SAM2 while highlighting its potential to drive innovation in precision livestock farming and similar real-world domains.

### 3.3. Comparison of the Activity Index Calculation with and Without Segmentation

**Figure 7:**
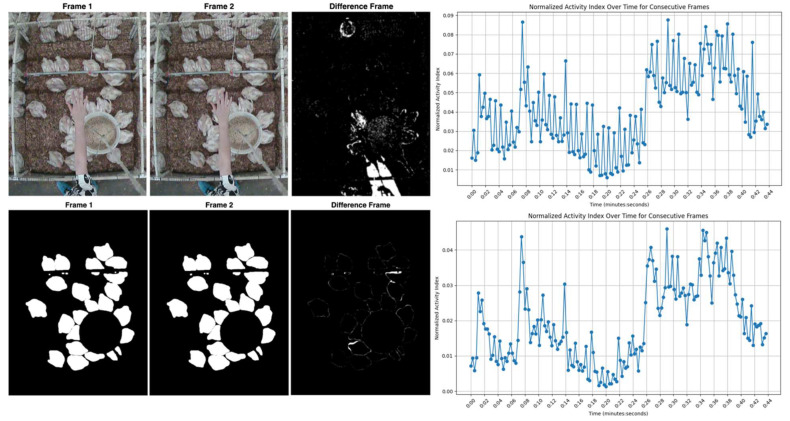
Effect of segmentation on the accuracy of the activity index: the top row shows the approach without segmentation, while the bottom row shows the approach with segmentation.

A total of 480 frames from week 4 recordings were analyzed to compare the results of activity index calculation with and without segmentation. As summarized in Figure 7, the segmented method produced lower and more consistent activity-index values (mean ± SD) relative to the unsegmented approach, indicating a reduction in background-induced noise.

A paired *t*-test revealed a significant difference (*p* < 0.01) between the two sets of activity-index measurements, demonstrating that removing non-essential background motion (e.g., human interference) meaningfully enhances the accuracy of the computed activity index. The activity index after segmentation was substantially reduced, with an average value of 3167.12 (mean absolute deviation of 2329.57), compared to 6302.64 (mean absolute deviation of 3744.55) recorded before segmentation (i.e., prior to normalization). Frames with noticeable external movement had higher activity indices under the no-segmentation approach, whereas the segmentation-based method isolated chicken-related motion, minimizing overestimation and producing a smoother time series. Unnormalized pixel change counts (3167.12 and 6302.64) were used for the statistical comparison of the activity index with and without segmentation to clearly illustrate the large difference in detected movement between the two approaches. The use of normalized values (0–1) would have compressed this range and made the contrast less apparent. For consistency with the described method, all final Activity Index values reported (e.g., see Figure 5) were normalized. These findings align with precision livestock monitoring studies, which have demonstrated that focusing on target subjects, such as chickens, reduces noise from extraneous interference by isolating them from distracting elements like moving litter, feathers, droppings, or human presence. This approach, particularly through image segmentation, improves data quality and tracking precision [17].

In practical applications, these results support the integration of segmentation as a pre-processing step in real-world poultry monitoring systems. By using segmented frames to calculate activity indexes, researchers can obtain more accurate, noise-free measurements that better reflect true animal activity. This refined approach can drive more effective, data-driven management decisions in precision livestock farming.

### 3.4. Determination of Optimal Sampling Ratio for Group Activity Assessment

**Figure 8:**
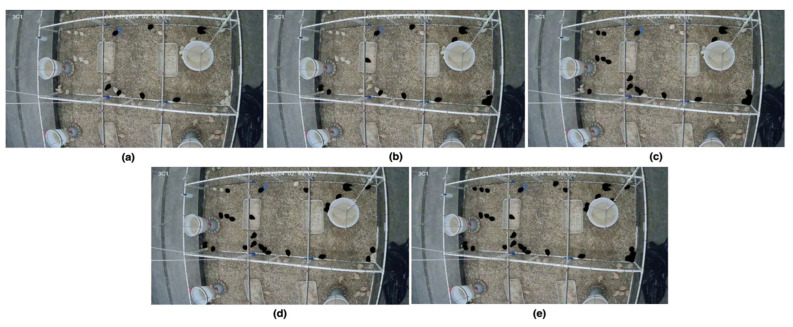
Example of five sampling initializations at week 1, comparing (**a**) 20%, (**b**) 40%, (**c**) 60%, (**d**) 80%, and (**e**) the entire flock (100%).

**Figure 9:**
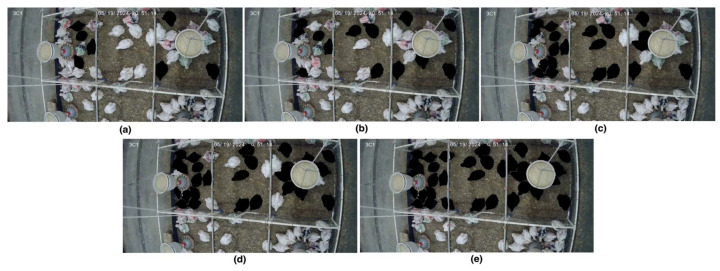
Example of five sampling initializations at week 4, comparing (**a**) 20%, (**b**) 40%, (**c**) 60%, (**d**) 80%, and (**e**) the entire flock (100%).

**Figure 10:**
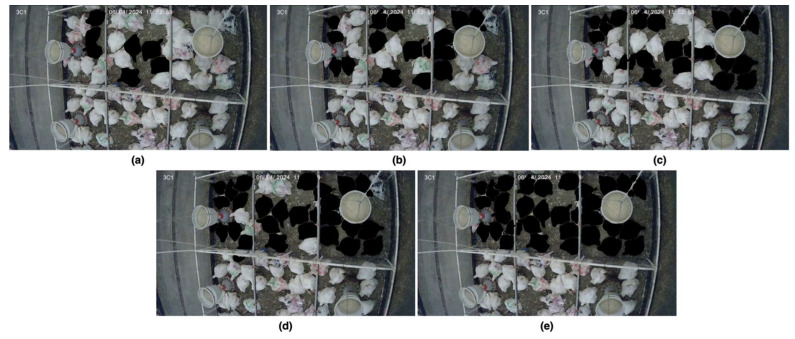
Example of five sampling initializations at week 7, comparing (**a**) 20%, (**b**) 40%, (**c**) 60%, (**d**) 80%, and (**e**) the entire flock (100%).

Four different sampling ratios (20%, 40%, 60%, and 80%) of the entire group were evaluated at three key broiler growth stages (weeks 1, 4, and 7). Six distinct initializations were selected from various regions of the pen for each percentage to minimize bias. Figure 8, Figure 9 and Figure 10 show different initializations for weeks 1, 4, and 7, respectively.

Table 2 summarizes the average *r* value between each representation’s activity index and the entire flock. For broilers in weeks 4 and 7, representations of 40% or more were highly correlated with the entire group (r ≥ 0.90), whereas in week 1, a subset of at least 60% was required to highly correlate with the entire group (r ≥ 0.93).

**Figure 11:**
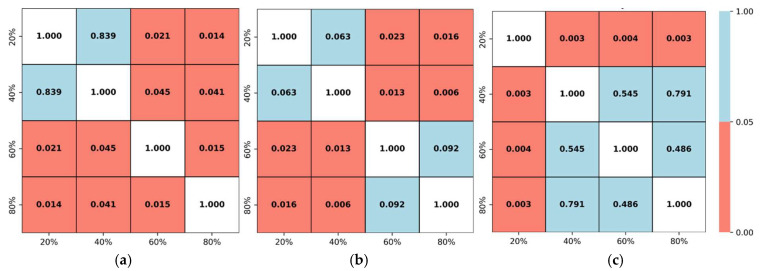
Comparative *p*-value heatmap across different representations at (**a**) week 1, (**b**) week 4, and (**c**) week 7.

To determine whether these representations also differ significantly from one another, *p*-values were computed and visualized in Figure 11 (heatmaps), with a significance level set at 0.05. Any pairwise comparison showing *p* < 0.05 was deemed significantly different, while *p*-values above 0.05 indicated no meaningful difference. At week 1 (Figure 11a), the 80% representation’s activity index was significantly different from that of all other subsets. Coupled with its high r value of 0.97, this finding underscored the need to track 80% of the flock during the first week to ensure a reliable movement indicator. In week 4 (Figure 11b), the 60% and 80% representations showed no significant difference from each other (*p* = 0.092) but differed significantly from both 20% and 40% (*p* = 0.006–0.023). Given that 60% alone achieved a high r value of 0.96 and was not significantly different from the 80% subset, the 60% emerged as a more cost-effective option to represent the entire group. Lastly, in week 7 (Figure 11c), 40%, 60%, and 80% exhibited no significant differences among themselves (*p* = 0.486–0.791), indicating that tracking 40% of the flock was sufficient, particularly given its high r value of 0.93 (Table 2).

The results demonstrate a clear trend that as broilers grew, the proportion of the flock required for accurate movement tracking decreased. This is likely due to the natural changes in flock behavior over time, where younger birds exhibited higher levels of individual movement variability, necessitating a larger sample size [40,41,42]. In contrast, older broilers exhibit more synchronized and predictable movement patterns, which allowed for a smaller subset of birds to sufficiently represent the entire flock [43,44].

From a practical standpoint, these results suggest that poultry management systems can significantly reduce tracking efforts by adjusting the sampling ratio based on bird age. Implementing an adaptive tracking strategy, in which a higher sampling ratio is used early in growth and gradually reduced over time, could optimize the efficiency of activity monitoring systems. This approach can help farms allocate computational resources more effectively, enabling real-time flock assessments without unnecessary data processing costs.

## 4. Conclusions

A user-friendly, open-source platform was developed to address key challenges in animal behavior monitoring by enabling the calculation of the activity index for individual and group-housed animals from video recordings. The SAM2 was integrated with a frame-subtraction approach, ensuring reliable segmentation and tracking without requiring extensive training or annotations. This segmentation-based method significantly reduced noise and interference, thereby enhancing the accuracy of activity-index calculations. The results suggested that 80% in week 1, 60% in week 4, and 40% in week 7 were sufficient to cover the entire group’s activity index. The computational burden was lowered by tracking fewer animals as broilers matured, while still maintaining a robust representation of overall flock activity.

Beyond broiler applications, the platform has the potential for deployment to other species, such as pigs, cattle, or laboratory mice, without necessitating specialized technical expertise, although performance may vary depending on species-specific characteristics and environmental factors. Data processing, segmentation, and activity-index visualization were consolidated into a single web-based interface, providing researchers with an accessible and efficient tool for analyzing animal behavior patterns. Consequently, a critical gap in the availability of free, online solutions for animal behavior research was filled, paving the way for broader automated analysis and further advancements in computational tools for automatic animal behavior analytics studies.

## Figures and Tables

**Table 1:** Segmentation performance of the Segment Anything Model 2 for segmenting broiler chickens at Weeks 1, 4, and 7.

Chicken Age	Evaluation Criteria (%)				
	Precision	Recall	F1 Score	IoU	Success Rate
Week 1	92.13	98.40	95.16	90.77	100
Week 4	94.42	98.29	96.29	92.85	100
Week 7	94.75	97.86	92.26	92.79	100

**Table 2:** Average Pearson correlation coefficients (r value) between each sampling ratio and the entire flock at different broiler growth stages (Weeks 1, 4, and 7).

Chicken Age	Sampling Ratio			
	20%	40%	60%	80%
Week 1	0.58	0.61	0.93	0.97
Week 4	0.74	0.90	0.96	0.98
Week 7	0.73	0.93	0.92	0.94

## Data Availability

The data presented in this study are available on request from the corresponding author. (the data are not publicly available due to privacy).

## References

[1] Bocaj E., Uzunidis D., Kasnesis P., Patrikakis C.Z. On the Benefits of Deep Convolutional Neural Networks on Animal Activity Recognition. Proceedings of the 2020 International Conference on Smart Systems and Technologies (SST).

[2] Oso O.M., Mejia-Abaunza N., Bodempudi V.U.C., Chen X., Chen C., Aggrey S.E., Li G. (2025). Automatic Analysis of High, Medium, and Low Activities of Broilers with Heat Stress Operations via Image Processing and Machine Learning. Poult. Sci..

[3] Tran D.-N., Nguyen T.N., Khanh P.C.P. (2022). An IoT-Based Design Using Accelerometers in Animal Behavior Recognition Systems. IEEE Sens. J..

[4] Elbarrany A.M., Mohialdin A., Atia A. Abnormal Behavior Analysis for Surveillance in Poultry Farms using Deep Learning. Proceedings of the 2023 Intelligent Methods, Systems, and Applications (IMSA).

[5] Chen C., Zhu W., Norton T. (2021). Behaviour recognition of pigs and cattle: Journey from computer vision to deep learning. Comput. Electron. Agric..

[6] Fuentes A., Yoon S., Park J., Park D.S. (2020). Deep learning-based hierarchical cattle behavior recognition with spatio-temporal information. Comput. Electron. Agric..

[7] Massari J.M., de Moura D.J., Nääs I.d.A., Pereira D.F., Branco T. (2022). Computer-vision-based indexes for analyzing broiler response to rearing environment: A proof of concept. Animals.

[8] Bloemen H., Aerts J., Berckmans D., Goedseels V. (1997). Image analysis to measure activity index of animals. Equine Vet. J..

[9] Aydin A., Cangar O., Ozcan S.E., Bahr C., Berckmans D. (2010). Application of a fully automatic analysis tool to assess the activity of broiler chickens with different gait scores. Comput. Electron. Agric..

[10] Li G., Li B., Shi Z., Zhao Y., Tong Q., Liu Y., Miglior F. (2020). Diurnal rhythms of group-housed layer pullets with free choices between light and dim environments. Can. J. Anim. Sci..

[11] Silvera A.M., Knowles T.G., Butterworth A., Berckmans D., Vranken E., Blokhuis H.J. (2017). Lameness assessment with automatic monitoring of activity in commercial broiler flocks. Poult. Sci..

[12] Kristensen H., Aerts J., Leroy T., Wathes C., Berckmans D. (2006). Modelling the dynamic activity of broiler chickens in response to step-wise changes in light intensity. Appl. Anim. Behav. Sci..

[13] Neves D.P., Mehdizadeh S.A., Tscharke M., de Alencar Nääs I., Banhazi T.M. (2015). Detection of flock movement and behaviour of broiler chickens at different feeders using image analysis. Inf. Process. Agric..

[14] Fernández A.P., Norton T., Tullo E., van Hertem T., Youssef A., Exadaktylos V., Vranken E., Guarino M., Berckmans D. (2018). Real-time monitoring of broiler flock’s welfare status using camera-based technology. Biosyst. Eng..

[15] Sengar S.S., Mukhopadhyay S. (2017). Moving object detection based on frame difference and W4. Signal Image Video Process..

[16] Li G., Chai L. (2023). AnimalAccML: An open-source graphical user interface for automated behavior analytics of individual animals using triaxial accelerometers and machine learning. Comput. Electron. Agric..

[17] Saeidifar M., Li G., Chai L., Bist R., Rasheed K.M., Lu J., Banakar A., Liu T., Yang X. (2024). Zero-shot image segmentation for monitoring thermal conditions of individual cage-free laying hens. Comput. Electron. Agric..

[18] Shams A., Becker D., Becker K., Amirian S., Rasheed K. Evolving Efficient CNN Based Model for Image Classification. Proceedings of the 2023 Congress in Computer Science, Computer Engineering, & Applied Computing (CSCE).

[19] Tan M., Chao W., Cheng J.-K., Zhou M., Ma Y., Jiang X., Ge J., Yu L., Feng L. (2022). Animal detection and clas-sification from camera trap images using different mainstream object detection architectures. Animals.

[20] Gandhi R., Gupta A., Yadav A.K., Rathee S. (2022). A novel approach of object detection using deep learning for animal safety. Proceedings of the 2022 12th International Conference on Cloud Computing, Data Science & Engineering (Confluence).

[21] Li G., Jian R., Jun X., Shi G. (2025). A Review of You Only Look Once Algorithms in Animal Phenotyping Applications. Animals.

[22] Campbell M., Miller P., Díaz-Chito K., Hong X., McLaughlin N., Parvinzamir F., Del Rincón J.M., O’COnnell N. (2024). A computer vision approach to monitor activity in commercial broiler chickens using trajectory-based clustering analysis. Comput. Electron. Agric..

[23] Ravi N., Gabeur V., Hu Y.-T., Hu R., Ryali C., Ma T., Khedr H., Rädle R., Rolland C., Gustafson L. (2024). SAM 2: Segment Anything in Images and Videos. arXiv.

[24] Qian R., Zhou L., Yu Y., Xu L. (2024). Accurate Beef Image Segmentation via Self-Prompting Guided Semantic Anything Model. Proceedings of the 2024 9th International Conference on Intelligent Computing and Signal Processing (ICSP).

[25] Noe S.M., Zin T.T., Tin P., Kobyashi I. (2023). Efficient segment-anything model for automatic mask region extraction in livestock monitoring. Proceedings of the 2023 IEEE 13th International Conference on Consumer Electronics-Berlin (ICCE-Berlin).

[26] Noe S.M., Zin T.T., Kobayashi I., Tin P. (2025). Optimizing black cattle tracking in complex open ranch environments using YOLOv8 embedded multi-camera system. Sci. Rep..

[27] Mathis A., Mamidanna P., Cury K.M., Abe T., Murthy V.N., Mathis M.W., Bethge M. (2018). DeepLabCut: Markerless pose estimation of user-defined body parts with deep learning. Nat. Neurosci..

[28] Romero-Ferrero F., Bergomi M.G., Hinz R.C., Heras F.J.H., de Polavieja G.G. (2019). idtracker.ai: Tracking all individuals in small or large collectives of unmarked animals. Nat. Methods.

[29] Dell A.I., Bender J.A., Branson K., Couzin I.D., de Polavieja G.G., Noldus L.P., Pérez-Escudero A., Perona P., Straw A.D., Wikelski M. (2014). Automated image-based tracking and its application in ecology. Trends Ecol. Evol..

[30] Ballerini M., Cabibbo N., Candelier R., Cavagna A., Cisbani E., Giardina I., Lecomte V., Orlandi A., Parisi G., Procaccini A. (2008). Interaction ruling animal collective behavior depends on topological rather than metric distance: Evidence from a field study. Proc. Natl. Acad. Sci. USA.

[31] Attanasi A., Cavagna A., Del Castello L., Giardina I., Melillo S., Parisi L., Pohl O., Rossaro B., Shen E., Silvestri E. (2014). Collective Behaviour without Collective Order in Wild Swarms of Midges. PLoS Comput. Biol..

[32] Cobb-Vantress (2021). Cobb Broiler Management Guide. https://www.cobbgenetics.com/assets/Cobb-Files/Broiler-Guide_English-2021-min.pdf.

[33] Girshick R. (2015). Fast R-CNN. arXiv.

[34] He K., Gkioxari G., Dollár P., Girshick R. Mask R-CNN. Proceedings of the 2017 IEEE International Conference on Computer Vision (ICCV).

[35] Redmon J., Farhadi A. (2018). YOLOv3: An Incremental Improvement. arXiv.

[36] Hinkle D.E., Wiersma W., Jurs S.G. (2003). Applied Statistics for the Behavioral Sciences.

[37] Kirillov A., Mintun E., Ravi N., Mao H., Rolland C., Gustafson L., Xiao T., Whitehead S., Berg A.C., Lo W.-Y. (2023). Segment Anything. arXiv.

[38] Sofiiuk K., Petrov I.A., Konushin A. Reviving Iterative Training with Mask Guidance for Interactive Segmentation. Proceedings of the 2022 IEEE International Conference on Image Processing (ICIP).

[39] Rother C., Kolmogorov V., Blake A. (2004). “GrabCut”: Interactive foreground extraction using iterated graph cuts. ACM Trans. Graph..

[40] Baxter M., O’cOnnell N.E. (2023). Large variation in the movement of individual broiler chickens tracked in a commercial house using ultra-wideband backpacks. Sci. Rep..

[41] Newberry R., Hall J. (1990). Use of pen space by broiler chickens: Effects of age and pen size. Appl. Anim. Behav. Sci..

[42] Weeks C., Danbury T., Davies H., Hunt P., Kestin S. (2000). The behaviour of broiler chickens and its modification by lameness. Appl. Anim. Behav. Sci..

[43] Bessei W. (2006). Welfare of broilers: A review. World’s Poult. Sci. J..

[44] van der Sluis M., de Klerk B., Ellen E.D., de Haas Y., Hijink T., Rodenburg T.B. (2019). Validation of an Ultra-Wideband Tracking System for Recording Individual Levels of Activity in Broilers. Animals.

